# Research on Theoretical Mechanism and Promotion Path of Digital Economy Driving China’s Green Development under “Double Carbon” Background

**DOI:** 10.3390/ijerph20010437

**Published:** 2022-12-27

**Authors:** Zhen Feng, Sainan Cheng, Guohua Qu, Yunlong Cui, Jiameng Ye

**Affiliations:** 1 School of Management Science and Engineering, Shanxi University of Finance and Economics, Taiyuan 030006, China; 2 School of Business Administration, Shanxi University of Finance and Economics, Taiyuan 030006, China; 3 School of Materials and Energy, University of Electronic Science and Technology of China, Chengdu 610054, China

**Keywords:** digital economy, green, environmental regulation, technical innovation

## Abstract

(1) Background: Under the background of building a new pattern of “double cycle” development, the green meaning of the digital economy is highly valued. The innovative feature of the digital economy is forming a new economic growth pole, and gradually becomes the driving force for China’s economic restructuring and green development; (2) methods: this paper empirically tests whether the digital economy can promote green development by using various econometric models based on panel dataset with 30 provinces from 2011 to 2019 in mainland China and measuring the development level of the digital economy and the greening index; (3) results: it is found that the digital economy can directly boost green development in greening degree of economic growth, resources and environment-carrying potential, and government policy support. The digital economy’s influence on green development has an inverted U-shaped trend; environmental control is an effective regulatory variable with a substitution effect on green development. With an obvious regional heterogeneity, the digital economy promotes green development; the digital economy can greatly affect green growth through technical innovation through mechanism analysis. The robustness test supports the above conclusion; (4) conclusions: the findings provide a foundation for multi-party policymakers to effectively formulate and implement policies for the digital economy that encourage green growth.

## 1. Introduction

China is experiencing a leap from seeking economic quantity and speed to seeking high-quality and sustainable development [1]. The law of economic development and the drastic changes in the general environment has changed the driving force of economic development from investment to innovation. The digital economy is now a new engine for high-quality economic growth because of its breakthrough innovation, positive externality, high growth, and rapid expansion. Accelerating digital development, creating new advantages for the digital economy, and fostering changes in production modes, lifestyles, and governance modes with digital transformation are all explicitly stated in the outline of the “14th Five-Year Plan.” It shows that the digital economy is the new engine and new driving force to promote the transformation and upgrading of China’s economic structure and green technological innovation. Green development is the development of efficient and economical resources, the development of a protected and clean environment, and the permanent and sustainable development of the economy and society. Economic level profoundly affects environmental quality, for instance, there is heterogeneity in the air pollution level in areas where different income groups live [2]. Digital technologies help innovate production technologies, improve resource consumption conditions, and empower traditional enterprises which have high energy consumption and high pollution to make green transformations. Emphasizing the layout of the digital economy has become an important channel to ensure high-quality economic development, promote green growth and ease the pressure of resource consumption [3]. With the concern of all sectors of society for ecology and green growth, corporate environmental behavior has become an important factor affecting green development. In particular, the potential impact of improper corporate behavior on the environment has become an urgent problem to be solved globally [4]. Promoting public participation in the third-party international environmental audit platform will help reduce information asymmetry, increase the driving force of corporate environmental governance, and thus help achieve green development [5]. According to the “Porter Hypothesis,” environmental regulation will motivate firms to innovate technologically, and strict environmental regulation can encourage enterprises to enter and quit market competition, as well as increase the effectiveness of enterprise monitoring [6]. If an environmentally responsible enterprise has advantages in reducing environmental governance costs, financial institutions will be more willing to provide financial support [7]. On the other hand, differential environmental regulation may bring about more serious pollution [8]. The government’s consumer guidance and penalties for polluting businesses can influence enterprises’ willingness to participate in third-party international environmental audits [9]. The effect of environmental regulation depends on the economic pressure of using natural resources [10]. How to achieve green economic growth through environmental regulation still requires seeking new power sources. The digital economy not only contains a lot of innovation opportunities but also has the rapid expansion of the platform economy, which can realize the win-win cooperation of stakeholders [11]. However, China’s digital economy is currently in a crucial stage of development, and regional digital economies’ capacity for development is uneven. Many “green potentials” remain untapped for the high-quality growth of a digital economy.

The rapid development of China’s economy has brought up serious environmental problems, hence the Chinese government has explicitly advocated the double carbon goals of “carbon neutrality” and “carbon peak”. Reduced industrial carbon emissions are an important aspect of achieving green development, and the double carbon target represents a significant challenge for industry. China’s total industrial green development level is low, but the trend is rising [12]. The digital economy is critical to reducing carbon emissions. In the long term, green technological growth and industrial structure upgrading will play a major role in affecting carbon emissions. The green integration of the digital economy and traditional industries is essential to reducing carbon emissions [13]. The promotion of the digital economy can effectively contribute to low-carbon sustainable urban development and will contribute much more if the degree of economic development reaches the threshold [14]. The digital economy affects carbon emissions through multiple pathways, and its development in most places can directly and effectively reduce carbon emissions [15]. The greater the depth of the digital transformation, the greater the influence of renewable energy on reducing carbon emissions. Seizing the advantage of the conditions and possibilities provided by digital transformation to achieve carbon neutrality will be critical to realizing green development [16]. Traditional industries may be transformed and upgraded to increase economic performance and decrease environmental pollution, while digital development, technological innovation, and industrial structure upgrades support the performance of a green economy [17,18]. Therefore, in the context of double carbon, the standards of green development will be more stringent, which will also bring new challenges and opportunities.

At present, against the background of the dual needs of developing the digital economy and green economy, on how to promote green development through the digital economy, it is vital to clarify the relationship between the digital economy and governmental environmental governance. Does the development of the digital economy, as it has an enhancing effect on high-quality economic development, have an impact on green development and in what specific aspects? Is there a nonlinear relationship between the digital economy and green development, and how can this relationship be measured? How do the mechanisms and laws between the digital economy and environmental regulations impact on green development? What are the differential impacts of the uneven development of the digital economy on green development in different regions of China?

Based on the questions raised above, this paper investigates the function mechanism and influence effect of the digital economy on green development through literature research and empirical testing, with practical implications for accelerating the development of the digital economy, playing its role in green development, and exploring its influence path.

## 2. Literature Review

Since Tapscott proposed the notion of “digital economy” in 1996, various communities have undertaken extensive studies [19]. The definition of the digital economy connotation emphasizes different historical stages, which has no unified standard. The early definition emphasized digital technology productivity, highlighting the digital technology industry and its market-oriented applications, such as the communication equipment manufacturing business, the information technology service industry, and the digital content industry [20]. The focus of research has gradually shifted to the interpretation of digital technology’s economic function and the transformation of production relations. The connotation of the digital economy contains the following four key components: (1) Digital information can be stored in a specific virtual carrier and reused [21]. (2) The Internet-created carrier carries market organizations and transmits digital information, such as sharing economy platforms, e-commerce platforms, and so on [22]. (3) Digital technology is the new generation of information technology capable of parsing and processing digital data, such as artificial intelligence, blockchain, cloud computing, big data, etc. [23]. (4) The new economic model and format are the results of digital technology innovation and integration with the traditional real economy, such as the new individual economy, unmanned economy, and so on [24]. This definition incorporates numerous perspectives, such as the application of technology, value creation, economic format, and strives to provide a comprehensive and detailed analysis of the digital economy. This paper studies a new economic and social development pattern–the digital economy following the agricultural and industrial economy. The digital economy uses digital knowledge and information as key factors of production, digital technology as the foundation, a modern information network as an important carrier, a digital platform as the primary medium, digital infrastructure as an important support, and the effective use of information and communication technology (ICT) as a driving force, all of which contribute to the optimization of economic structure and high-quality development [25].

The measurement of the digital economy comes from the following areas. First, the entire extent of the digital economy in certain areas or industries is measured using numerous studies released by research institutions or departments. The Digital Economy and Society Index (DESI) is a key indicator used by the European Commission to measure the digital progress of member states. Thirty-one secondary indicators of broadband access, human capital, Internet adoption, digital technology adoption and the extent of digital public services released by the “Digital Economy and Social Index (DESI) 2022” were calculated to derive the DESI for each EU country. The U. S. Department of Commerce calculates the size of the digital economy by analyzing the economic impact of digitalization. “Measuring the Digital Economy: A New Perspective”, published by the Organization for Economic Cooperation and Development (OECD), constructs the digital economy system through 38 indicators, including broadband penetration, Internet users, mobile data communications, Internet development, network connection prices, ICT devices and applications, and cross-border e-commerce, etc. China Center For Information Industry Development (CCID) Consulting Digital Economy Industry Research Center has initiated the “4 + 3 + N” evaluation system of the “DEDI Index” around the development of China’s regional digital economy. Eight first-level indicators, including digital foundation, digital industry, digital integration, digital governance, main vitality, innovation impetus, capital heat, and public participation, were selected and refined into 55 second-level indicators. The indicator data comes from the “2022 China Digital Government Application Development Research Report” released by the Digital Economy Industry Research Center of CCID Consulting. The White Paper on the Development and Employment of China’s Digital Economy (2019) released by the China Academy of Information and Communication Research proposed that the development of the digital industry is mainly reflected in terms of industrial scale, network infrastructure, and revenue capacity [26]. The second is using statistical data to build an evaluation index system, which earlier started from the Internet and ICT technology [27], and declared that the digital economy is based on the effect of the Internet. Subsequently, the influencing factors were expanded to include digital transactions [28], digital economy carriers [29], the digital literacy of residents [30], and the digital economy environment [31], where the indicator data mainly come from the China Statistical Yearbook 2016 to 2019 published by the National Bureau of Statistics of China, China City Statistical Yearbook, Digital China Index Report, panel data of 30 provinces in mainland China (excluding Hong Kong, Macao, and Taiwan as well as Tibet) from 2013 to 2017, etc. Dimensions, such as the establishment of a multi-dimensional index system including digital infrastructure, digital industrialization, digital applications, digital market, and labor force [3]. Although the measurement methods and index designs of the digital economy are different at present, there is no clear and unified definition yet, and the influence of the digital economy is constantly changing with time and space. The spillover effect of the digital economy should also be included in the evaluation index system. Therefore, the measurement of the digital economy should integrate the influences of all dimensions, paying attention to both digital technology itself and digital infrastructure, as well as the impact of production innovation and industrial structure brought by digitalization, and the social foundation of the digital economy development.

The germination of the idea of green development can be traced back to the Blue Book of Green Economy published in 1989, which proposed the establishment of an “acceptable economy” [32]. Economic development should be based on the social environment and resource conditions, and it is generally considered sustainable development to pursue green development of economic activities within the tolerable range of natural resource consumption and environmental pollution [33]. Green development is supported by environmental protection and resource-saving technologies [34]. Man-made capital, rather than natural environmental capital, can increase material resource efficiency and the growth mode of high energy consumption and pollution [35]. Green development integrates environment, economy, politics, and culture [36], while also emphasizing the integrity, systemic, and coordinated interaction of the economic, social, and natural systems [37].

There are two main methods to measure the performance of green development. One is the index system method, which designs an evaluation index system according to the influence sources of green development concerns. For example, China’s Green Development Index measures the greening degree of economic growth, the carrying potential of resources and the environment, and the level of support for government policies [38,39]. The other is the derivative model of DEA and its technical optimization. Based on the DEA model, non-fossil energy consumption is set as an input index, and economic development and carbon emissions are taken as output indexes to measure green development efficiency [40]. It is found that technological progress is the main driving force for the high-quality promotion of the Yangtze River Economic Belt [41].

This paper summarizes the impact of the digital economy on green development in terms of direct and indirect effects. Regarding direct impacts, the role and economic complexity of ICTs in the digital economy are critical for controlling environmental unsustainability and developing policies to address ecological issues. ICT exports, economic complexity, and economic growth raise the intensity of the ecological footprint, while ICT imports, R and D, and trade help G7 economies minimize their ecological footprint [42]. It is pointed out that with the development of the digital economy, the greening of the economy will increase first and then decrease, while the energy intensity will decrease first and then increase [30]. It shows that the “energy rebound effect” leads to the “inverted U-shaped” constraint of the digital economy on greening. The digital economy has a non-linear influence with an increasing marginal effect on China’s industrial green total factor productivity [43]. How to promote the sustainable economic development of emerging countries through the opportunities brought by demographic dividends, digital economy, and energy efficiency is also studied [44]. The demographic dividend and digitalization are found to stimulate sustainable economic growth in all quartiles. There are positive correlations among urbanization, capital formation, and industrialization, while energy intensity and economic sustainability are negatively related to sustainable economic growth. Realizing the demographic dividend opportunities and utilizing digital innovation in the energy sector will contribute to economic performance.

As for indirect impacts, digital reforms and information technology have greatly affected Chinese agriculture, rural areas, and farmers. Internet popularization and digital technology have promoted the sustainable development of China’s agriculture [45]. Green technology innovation is the transmission path for digital finance to affect China’s energy and environmental performance. The financial and environmental supervision of the Chinese government can strengthen the role of digital finance in promoting energy and environmental performance [46]. It is found that the digital finance brought by the Internet revolution develops more rapidly in backward areas, and significantly improves the economic level of rural low-income groups [47]. It is pointed out that technological progress and environmental regulation are helpful to promote China’s industrial green total factor productivity and the strong government environmental regulation raises the threshold of environmental conditions for foreign investment entry [48]. It is found that the digital economy has a positive impact on the transition of renewable energy consumption and generation, and government governance has a positive impact on the relationship between the digital economy and the energy transition [49]. The consumption structure of renewable energy will expand by 0.021% for every 1% growth in the digital economy, while the power-generating structure of renewable energy will increase by 0.106%. Furthermore, the digital economy promotes renewable energy transformation by stimulating the government’s governance capabilities. The digital economy promotes green total factor productivity in a “structural” way, while industrial digitalization and digital industrialization are the long-term driving forces [29]. The digital economy is progressively becoming an essential driving force for regional low-carbon development by constructing China’s digital economy evaluation system [31]. The major channels of influence are environmental governance, technical innovation, and industrial structure modernization, with industrial structure upgrading playing a larger intermediate role than technological innovation.

## 3. Hypothesis

Based on the above discussion, this paper will demonstrate the basic function, influence mechanism, and transmission path of the digital economy to green development, and put forward the corresponding research hypothesis.

Among a series of economic activities caused by the digital economy, the digital economy has the characteristics of rapid growth, positive externality, frequent subversive innovation, optimization of production resource allocation, improvement of high-energy consumption growth mode, etc., which effectively expands the application scenario of digital technology, breaks the boundary of digital economy development, and alleviates the resource supply and demand obstacles in the green development of the economy. The proper integration of the digital economy and real economy also breaks through the fetters of digital economy development in geographical scope and promotes green development in various ways. The digital economy, as a new economic form, uses digital knowledge and information as fundamental production components, which is directly beneficial to the vitality of urban green growth [50]. The fast-growing digital economy sector contributes to green growth by squeezing out high-polluting industries, as it is concentrated in the ICT sector, which is less harmful to the environment [51]. The driving role of digital technology in the quality development of emerging industries further inspires the need for digital transformation and sustainable green economy development. The digitalization of enterprises can also be enhanced through the application of ICT, which can increase the use of information technology and productivity in the production departments [52]. The new generation of digital technology is gradually becoming a new dividend that leads to green development and pollution reduction with a diminishing marginal effect, in comparison to the conventional land and energy dividends [53]. The effective use of ICT with modern information networks as the key carrier in the digital economy is more indicative of the efficiency improvement and economic structure optimization. At the same time, the development of digital technology, ICT technology, and the Internet may also have “Metcalfe’s law”, that is, the value of the network increases at the square of the number of users. Therefore, the influence of the digital economy on green development may show a nonlinear characteristic of increasing marginal effect. Therefore, the following research hypotheses are proposed.

**H1.**  *Digital economy can directly promote green development*.

**H2.**  *Digital economy has an inverted U-shaped influence on green development*.

Environmental regulation can improve environmental quality and reduce disease and mortality. In 1990, the United States promulgated the Clean Air Act Amendment (CAAA), which brought about a gradual reduction in air pollution. CAAA encourages local regulators to target the dirtiest areas at first, so as to form a heterogeneity in the incidence of air quality improvement, which is beneficial to low-income families [54]. Labor costs and environmental regulations are significantly lower in developing countries than in developed countries, creating a competitive advantage in the global marketplace [55]. To cope with environmental regulation or offset the productivity decline caused by pollution, the investment and emission reduction measures taken by enterprises will have an impact on the capital productivity of enterprises [56]. Digitalization not only plays an important role in environmental improvement and innovative development [57] but also can promote the development of a green economy and sustainable economy [58,59]. The digital economy has been fully developed in the market environment. However, as far as enterprises are concerned, they may face challenges of profitability and social responsibility in the course of their operation, and relying solely on market forces may not be able to solve the external uneconomical problems of enterprise production. Thus, the impact of environmental policies on green economic growth has become an important concern. Between 1990 and 2008, the hidden pollution tax faced by manufacturers in the US manufacturing industry doubled. Changes in various environmental regulations, rather than changes in productivity and trade, are the main reasons for the reduction of emissions [60]. The government’s introduction of low-carbon policies, management of environmental pollution control, and layout of carbon emission reduction projects have become feasible ways to achieve green and sustainable development. The implementation of carbon constraints on economic growth is an inherent requirement for China and the world to achieve sustainable development in the future. By forcing enterprises to innovate and adopt cleaner production technologies, government environmental regulation promotes the transformation and upgrading of industrial structures, which is conducive to the improvement of green economic efficiency. During the period from 2012 to 2017, green entrepreneurship supported by the Saudi government’s commitment contributed positively to the economic, social, and environmental components of sustainable development [61]. The use of digital technologies has greatly facilitated technical and managerial convenience in government environmental regulation and governance. Through the application of digital technology, it can promote multi-sectoral information communication and multi-agent coordination and command, effectively improve the government’s environmental pollution supervision level for enterprises and the government’s propaganda ability for the public to protect resources and the environment and play a role in environmental governance and protection [62]. Therefore, the following hypothesis is made.

**H3.**  *Environmental regulation plays a regulatory role between the digital economy and green development*.

The reorganization of manufacturing elements is considered as a source of innovation. The primary strategy for advancing green growth should be thought of as technological advancement and innovation [63]. The digital economy has brought significant enabling effects, promoted the transformation of products, business types, and industrial models, and opened up new development paths and feasible spaces for effective breakthroughs in innovation activities [64]. The digital economy has realized technological innovation with high efficiency, low cost, and less resource consumption. Digital technology itself has technical characteristics. In the production process, digital technology is based on new production factors such as knowledge, information, and data. Under the action of technicians, digital technology fully integrates with traditional production factors and new production factors, realizes the full flow and efficient allocation of production factors, promotes the close combination and coordinated development of digital technology and production, further enhances the application scope of digital technology [65], enhances its influence ability, and promotes the combination and matching of digital technology with other innovative factors and innovative subjects. In this process of production and activities, a series of economic activities in which digital technology participates becomes the core of the digital economy. Knowledge evaporation and technology diffusion support corporate green technology acquisition and regional technology upgrading, both of which enhance the quality of economic development. As a result, hypothesis H4 is proposed.

**H4.**  *Technological innovation may play an intermediary role between the digital economy and green development*.

**Figure 1:**
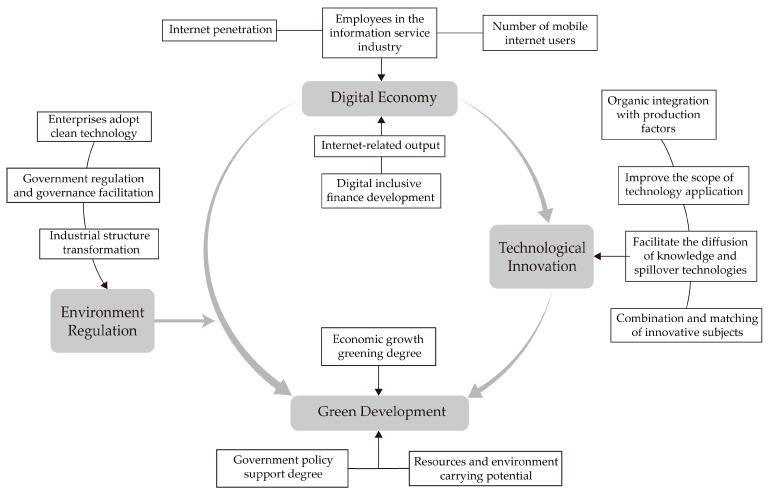
The theoretical framework of the digital economy driving green development.

Based on the analysis, a theoretical framework diagram of the digital economy driving China’s green development is drawn, as shown in Figure 1.

This article makes the following innovations and contributions: First, based on the existing literature research and economic theory, it proposes the theoretical hypothesis that the digital economy affects the green development of the economy. Concerning the existing research results, this paper outlines statistics and measurements on the development of the digital economy and green development in China’s provinces through the index system method, and expounds on and tests the role of direction and influence degree of the digital economy composite index on economic greening and its sub-dimensions. Second, to consider the role of government oversight in the impact of the digital economy on green development, and study the effect of environmental regulation. Third, to study the influence path of the digital economy on economic greening, an intermediary effect model is put forward for empirical analysis. The study’s conclusions offer a theoretical basis and practical reference for formulating policies to support the synergistic growth of digitalization and greening.

## 4. Materials and Methods

### 4.1. Model Setting

#### 4.1.1. Fixed Effect Panel Data Model of the Digital Economy Affecting Green Development

To measure the effectiveness of the digital economy on green development, a fixed effect model is constructed, using the Formula (1) [27].

*GREEN_it_* = a + b_1_ *DIG_it_* + b_3_ *CONTROL_it_* + u_i_ + r_t_ + e_it_ (1)

In formula (1), “a” is the constant term of the regression of *DIG_it_* to *GREEN_it_*, “b_1_” is the regression coefficient of *DIG_it_* to *GREEN_it_*, “b_3_” is the regression coefficient of *CONTROL_it_* to *GREEN_it_*, i means different provinces, t denotes different years, *GREEN_it_* indicates green development level, *CONTROL_it_* includes a series of control variables such as INR, MAR, URB, SCAL, OPEN, and HUM. The u_i_ and r_t_ represent individual and time-fixed effects, respectively, and e_it_ are random disturbance terms.

#### 4.1.2. Nonlinear Effect Test Model of the Digital Economy Affecting Green Development

To test that the impact of digital economy development on the greening of the economy is likely to show an inverted U-shaped relationship, the quadratic term of the digital economy is constructed [30]. In Formula (2), the quadratic term is used to measure the digital economy, and the meanings of other variables and parameters are the same as those in formula (1). *DIG*^2^*_it_* is used to measure the digital economy. “a” is the constant term of the regression, “b_1_” is the regression coefficient of *DIG_it_* to *GREEN_it_*, “b_2_” is the regression coefficient of the quadratic term *DIG*^2^*_it_* to *GREEN_it_*, “b_3_” is the regression coefficient of *CONTROL_it_* to *GREEN_it_*.

*GREEN_it_* = a + b_1_
*DIG_it_* + b_2_
*DIG*^2^*_it_* + b_3_
*CONTROL_it_* + u_i_ + r_t_ + e_it_ (2)

#### 4.1.3. Moderating Effect Test Model

To check the regulatory effect of environmental regulation on the development of the digital economy in the process of economic greening, and build a model as shown in Formula (3) [66]. *DIG_it_* * *REG_it_* is the interactive item of the digital economy and environmental regulation, indicating the regulatory effect. When the coefficient is less than 0, it indicates a substitution effect; if it is greater than 0, it indicates a complementary effect. Other variables have the same meanings as in Formula (1). “a” is the constant term of the regression, “b_1_” is the regression coefficient of *DIG_it_* to *GREEN_it_*, “b_2_” is the regression coefficient of *REG_it_* to *GREEN_it_*, “b_3_” is the regression coefficient of the interactive item *DIG_it_* * *REG_it_* to *GREEN_it_*, “b_4_” is the regression coefficient of *CONTROL_it_* to *GREEN_it_*.

*GREEN_it_* = a + b_1_
*DIG_it_* + b_2_ *REG_it_* + b_3_
*DIG_it_* * *REG_it_* + b_4_
*CONTROL_it_* + u_i_ + r_t_ + e_it_ (3)

#### 4.1.4. Intermediary Effect Test Model

Models are built in accordance with Formulas (4) and (5) to check the intermediary effect of technological innovation on the impact of digital economy development on the process of the economy’s greening [67]. In Formula (4), *INN_it_* indicates the level of technological innovation and development, “a” is the constant term of the regression, “b_1_” is the regression coefficient of *DIG_it_* to *INN_it_*, and “b_2_” is the regression coefficient of *CONTROL_it_* to *INN_it_*. In formula (5), “a” is the constant term of the regression, “b_1_” is the regression coefficient of *DIG_it_* to *GREEN_it_*, “b_2_” is the regression coefficient of *INN_it_* to *GREEN_it_*, “b_3_” is the regression coefficient of *CONTROL_it_* to *GREEN_it_*. The meanings of other variables are the same as in Formula (1).

*INN_it_* = a + b_1_
*DIG_it_* + b_2_
*CONTROL_it_* + u_i_ + r_t_ + e_it_ (4)

*GREEN_it_* = a + b_1_
*DIG_it_* + b_2_
*INN_it_* + b_3_
*CONTROL_it_* + u_i_ + r_t_ + e_it_ (5)

### 4.2. Variables and Data

#### 4.2.1. Explained Variables

In the existing research, the index system for measuring the green index pays more attention to resources and the environment, while the development index mainly focuses on economic growth. To highlight the perfect combination of green and development, the indicators and methods of the China Green Development Index Annual Report [38] are used to evaluate the green development, economic development, and resource and environmental protection of 30 provinces (cities, districts) in mainland China (excluding Tibet, Hong Kong, Macao, and Taiwan) from 2011 to 2019, to fully reflect the core content of the combination of green and development; that is, to focus on the degree of green development of industrial development, the degree of resource and environmental protection, government planning and attention, etc. The index system includes the Greening Index (GREEN), as well as the economic growth greening degree (ECO), resources and environment carrying potential (ENV), and government policy support degree (GOV) of secondary indicators [39].

#### 4.2.2. Core Explanatory Variables

To synthesize the relevant measurement indicators of the digital economy in existing studies [68,69], and the availability of data, the index system of the digital economy composite index (DIG) is established, as shown in Table 1. By using the improved entropy method, the index of the digital economy is weighted and the comprehensive index is calculated [70].

#### 4.2.3. Adjustment Variables

The influence of environmental regulation on green development may have two effects. First, environmental regulation increases the technical management cost in enterprise production and squeezes the investment in technological innovation and the introduction of human capital, which may hinder technological innovation and be detrimental to green development. On the other hand, environmental regulations force enterprises to adopt more advanced technologies and production methods, promote scientific and technological innovation and industrial upgrading, and raise green development levels. In this paper, the comprehensive index of environmental regulation (REG) is obtained by standardizing industrial wastewater discharge, industrial sulfur dioxide emissions, and industrial smoke (powder) dust emissions [71]. The lower the value is, the less pollution is emitted. Environmental regulations become more stringent as a result of a stronger political climate about pollution control.

#### 4.2.4. Intermediary Variables

The positive effect of the digital economy on social development is conducive to improving the overall level of green development. Technological innovation can improve the utilization efficiency of factor resources, reduce energy consumption, promote economic growth, achieve energy conservation and emission reduction, thus enhancing the degree of green development. This paper chooses the level of technological innovation and development (INN) as the intermediary variable. The proportion of R and D expenditure (100 million yuan) in the GDP of industrial enterprises above the designated size indicates the level of technological innovation [72].

#### 4.2.5. Control Variables

Since the factors affecting green development are complex and diverse, a series of control variables are added to make the measurement model more robust. The measurement of industrial structure rationalization (INR) draws lessons from research [73]. A Theil index of 0 is the equilibrium state of the economy; otherwise, the industrial structure deviates from the equilibrium state. The marketization index (MAR) comprehensively reflects the marketization degree of a region. The data are derived from the report on China’s Sub-provincial Marketization Index (2019) compiled by other scholars [74]. On the one hand, urbanization (URB) is beneficial for upgrading industrial structures as well as the efficiency of economic growth [75]. On the other hand, it also increases the consumption of resources and environmental pollution, which may hurt the efficiency of the green economy. Urbanization is measured by the ratio of the urban population to the total population, in which the total population is the resident population. The macro-control function of local government on the market has an important influence on the quality of economic development, and fiscal spending is the main measure taken by the government. Adopting the proportion of fiscal expenditure to GDP (SCAL) represents the scale of government [76]. Opening to the outside world (OPEN) can affect the quality of regional economic development through technology spillovers and “pollution shelters” [77]. The ratio of foreign investment to GDP is used to indicate the level of opening up. The promotion of human capital (HUM) is helpful for absorbing and applying foreign advanced technologies, researching and developing new technologies, promoting regional technological innovation, reducing energy consumption, and playing an important role in the quality of economic development. The level of human capital is measured by the average years of education at the provincial level in this article [78].

China Environmental Statistical Yearbook and China Energy Statistical Yearbook, as well as the statistical yearbooks for each province mainly contained information of the variables, where individual missing data are supplemented by interpolation. The descriptive statistics for the main variables are shown in Table 2.

## 5. Discussion

### 5.1. Basic Characteristics of Facts

**Figure 2:**
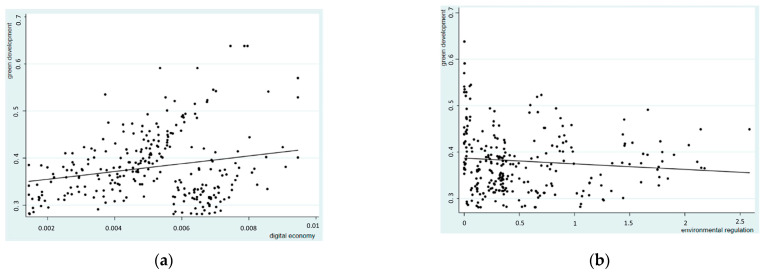
Linear fitting of the digital economy and greening as well as environment regulation to greening: (**a**) Linear fitting of digital economy and greening; (**b**) Linear fitting of environmental regulation to greening.

Figure 2a depicts a linear fit between the digital economy and green development, indicating that the level of green development steadily grows as the digital economy improves. Figure 2b represents the linear fitting trend of environmental regulation and green development. As the degree of environmental regulation degree increases, that is, the more industrial wastes are discharged, the greater is the environmental pollution degree, and the lower is the local green development level.

**Figure 3:**
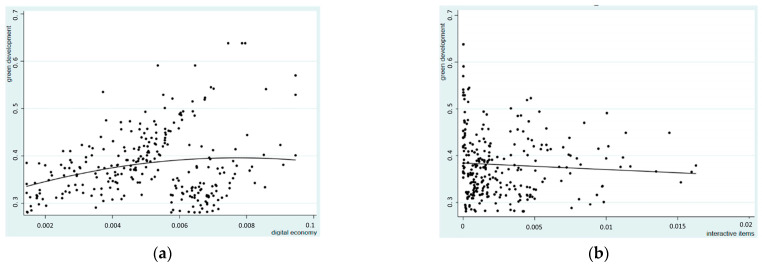
Quadratic linear fitting of the digital economy and greening as well linear fitting of interactive items to greening: (**a**) Quadratic linear fitting of digital economy and greening.; (**b**) Linear fitting of interactive items to greening.

Figure 3a shows the quadratic fitting curve of the relationship between the digital economy and green development, with the horizontal axis representing the digital economy development level, and the vertical axis indicating the index of green development. The two variables have a tendency to exhibit a broad mouth inverted U-shape. It suggests that as the digital economy growth level rises over the research sample period, green development would rise first and then progressively decline after reaching a specific level. Figure 3b shows the linear fitting trend of the interaction term between the digital economy and environmental regulation on green development. The horizontal axis represents the interaction between the digital economy and environmental regulation, and the vertical axis is the greening index, which shows a weak negative correlation. It means that the regulatory effect of environmental regulation on the digital economy is expressed as the substitution effect. How to achieve sustainable economic development through a digital economy and green new policy still needs to be studied in depth.

### 5.2. Estimated Results of the Digital Economy’s Impact on Green Development

#### 5.2.1. Control Variables

Table 3 reports the direct impact of the digital economy on green development. In columns (1), (2), and (4), the regression coefficients of the digital economy on green development are positive, and passed the 1% significance level test, assuming that H1 has been verified. It shows that the digital economy has a significant impact on green development, the greening degree of economic growth, and government policy support, that is, the development of the digital economy can promote green development, although the degree of impact varies. The regression coefficient of the digital economy on green development is 16.3204, indicating that for every 1% change in the digital economy index, the level of green development increases by 16.3204%, while the regression coefficient of the greening degree of economic growth is 5.44, which indicates that production has a significant impact on resource consumption and environment. The regression coefficient of government policy support is 15.8743. For every 1% increase in a digital economy development capacity, the government policy support will increase by 15.8743%. It shows that social organizers have a strong level and strength in dealing with the contradiction between resources, environment, and economic development, that is, the digital economy empowers the government to have a more profound impact on green investment, infrastructure, urban management, and environmental governance. On the contrary, column (3) of Table 3 shows that the regression coefficient of the digital economy on the carrying potential of resources and environment is −3.8639, indicating that with the improvement of the digital economy, the carrying potential of natural resources and environment is weakened. At present, all fields of the digital economy and industrial production have accelerated the pace of integration. In the process of integration, the digital economy has reconstructed the traditional productivity system which relies on the expansion of production factors and alleviated the economic effects of high input, high energy consumption, high pollution, and low benefit of traditional production methods. Gradually, the digital economy has formed a stronger driving force in promoting sustainable economic development.

Among the control variables, the scale of government and the level of opening up have significantly positive effects on green development, while the rationalization of industrial structure and marketization have no significant negative effects on green development, while urbanization and human capital have significant negative effects. It has the same effect on the greening degree of economic growth. The scale of the government has significantly promoted green development. On the one hand, the part of government infrastructure construction and environmental governance expenditures used for the quality of economic development has played a positive role. On the other hand, fiscal expenditure is an important means of government macro-control. Appropriate use of regulatory tools can make up for market failures, improve the efficiency of factor flow and resource allocation, and enhance the development level of the digital economy. By significantly enhancing the level of green development, opening up shows that the flow of knowledge, technology, and production factors can promote technological innovation, effectively enhance economic externalities and promote the greening of circulation links in the process of attracting investment.

The negative impact of industrial structure rationalization on green development is not significant, mainly because the application scenarios of digital technology are not ubiquitous, and some industries with low development quality have not yet been fully digitized. Therefore, although the digital economy can advance the upgrading of industrial structures, the rationalization of industrial structures is not possible. Against the background of the digital economy, the quality of regional investment may not be high enough, and it is more concentrated in low-end industries. Although it contributes to the rapid development of the economy in China, it is not conducive to the upgrading of the industrial structure. Marketization has a negative impact on green development, which may be because regional marketization development includes multi-dimensional indicators such as product market, factor market, and intermediate market. Under the condition of insufficient development, it may be detrimental to the development efficiency of the green economy. It has a significant negative impact on the green development of urbanization because the traditional urbanization model in China is mainly diseconomies of scale and high environmental pollution caused by population change. Moreover, with the deepening of industrialization accompanying urbanization development, the damage of the ecological environment caused by production activities is aggravated by rapid economic growth, which leads to the contradiction between economic and social development and the ecological environment. The influence of the human capital level on green development is significantly negative. With the increase of per capita education level, people’s awareness of environmental protection and sense of social responsibility will be enhanced, and attention will be paid to resource conservation and environmental protection. The quality of human capital is a long-term influencing factor to promote green development. At present, the level of human capital in some regions of China still has much room for improvement.

#### 5.2.2. Direct Effects of Environmental Regulation on Green Development

Table 4 reports the direct impact of environmental regulation on the digital economy. In the first, second, and fourth columns, the regression coefficient of environmental regulation on green development is 0.0465, which is significantly negative, indicating that for every 1% increase in pollution emissions, the level of green development decreases by 0.0465%. It shows that environmental regulation has a significant inhibitory effect on green development, greening degree of economic growth, and government policy support; that is, the lower the level of environmental regulation, the higher the degree of environmental pollution and the harder for government to solve problems between resources and green development. In column (3) of Table 4, the regression coefficient of environmental regulation on the carrying capacity of resources and environment is 0.0111, indicating that environmental regulation has improved the carrying capacity of natural resources and the environment. Combining the three dimensions, it can be considered that the effect of environmental regulation on the environmental carrying capacity of natural resources offsets its influence on the greening degree of economic growth and the government’s efforts to solve green development issues in the statistical results. Under the new development pattern, the market plays a major role in resource allocation, although relying on market forces alone to resolve the contradiction between ecological and environmental pollution and stable economic growth is still slightly insufficient. The government needs appropriate environmental regulations to play a regulatory role, further promote sustainable development and realize the coordinated evolution of economic growth and green development. The measures taken by the government include environmental policies and financial expenditures, which mainly affect the production activities of enterprises and regional industrial adjustment through supervision and optimization of resource allocation. Enterprises are required to pay attention to technological greening and environmental pollution issues, improve traditional technical means and enhance green output.

#### 5.2.3. Non-Linear Effect of the Digital Economy on Green Development

Table 5 summarizes the regression results of formula (2) and the nonlinear effect of the digital economy on green development. In column (1), the regression coefficient of the digital economy on green development is 73.7503 and the regression coefficient of the quadratic term of the digital economy on green development is −6.4 × 10^3^ ***, which shows that the impact of the digital economy on green development has an inverted U-shaped trend and is significant at the 1% level, and hypothesis H2 is verified. It shows that as the level of the digital economy increases, the level of green development first increases and then gradually decreases. The possible reason is that the digital economy, as a direct manifestation of digitalization, essentially reflects the level of regional economic growth brought by enterprises and industries. Digitalization can not only greatly reduce the cost of enterprise information search and logistics in the industrial chain, but also help to obtain the spillover effects brought by technology diffusion in regional innovation network systems, which can lead to green production and sustainable development of enterprises. However, when digitalization develops to meet the needs of enterprise production and technological innovation, if the development of the digital economy is further enhanced, blind investment and scale expansion may instead bring about diminishing marginal effects, resulting in a decrease in the marginal output of green development.

In columns (2) and (4) of Table 5, the digital economy also has a significant inverted U-shaped influence trend at the level of 1% on the greening degree of economic growth and government policy support. Although the degree of influence is close, there are still differences. The possible reason may be that when the digital economy develops at a low level, it can significantly improve the green development of the economy. However, with the enhancement of the allocation and development ability of the digital economy, the mismatch of information transmission, software service-related employees, and so on brought by it or the difficulty of being fully utilized will make it more difficult to promote green development. When government policy support is too high, it may lead to idle public assets and inefficient governance, which makes it difficult to continue the positive impact of the digital economy on government policy support. In column (3) of Table 5, the influence trend of the digital economy on the carrying potential of resources and environment shows a U-shaped change, indicating that as the development capacity of the digital economy increases, the carrying potential of resources and environment will first decline and then gradually increase. The possible reason is that the infrastructure investment, high-tech requirements, and human capital participation in the initial stage of the digital economy will bring about a concentrated scale of resource consumption, which will rapidly reduce the carrying capacity of resources and the environment. However, as the scale of the digital economy escalates, green production and environment-friendly production can increase the carrying capacity of resources and the environment, and guarantee the green development of the economic environment.

#### 5.2.4. Moderating Effect of the Digital Economy and Environmental Regulation on Green Development

The moderating effect of digital economy and environmental regulation on green development was tested using Formula (3). The regression results show that there is a substitution effect, as seen in Table 6. The data in the table indicates that hypothesis H3 is verified. Column 1 of Table 6 shows that environmental regulation inversely regulates the impact of digital economy on green development. The regression coefficient of the digital economy on green development is 18.7761, which means that for every 1% increase in the digital economy, the level of green development will increase by 18.7761%. The coefficient of the interaction term between the digital economy and environmental regulation is −0.8661, indicating that pollution emissions inhibit the promotion of digital economy to green development. The possible reason is that the initial development of the digital economy requires a large amount of information infrastructure investment and construction is needed, and the related technologies are generally concentrated ones with high demand for electric power. High-intensity resource consumption will cause high pollution to the environment, which will make it difficult to promote the development of a green economy in the short term. The economies of scale played by the digital economy can reduce the cost of green innovation and energy consumption. However, when the degree of environmental pollution is severe, the development of the digital economy in some areas will have great difficulties. In addition, due to the pressure of resources and the environment, it is impossible to rely on the development of the digital economy to improve the efficiency of green development. Columns (2) and (4) show that the digital economy’s improved comprehensive capability can boost greening of economic growth and government policy support. The results in column (3) reveal that the moderating effect of environmental regulation is offset by the influence of the digital economy on green development. However, the digital economy can still have a beneficial influence on green growth since environmental regulation can increase the carrying capacity of the resource environment.

### 5.3. Regional Heterogeneity Test of the Digital Economy on Green Development

In view of the unbalanced regional development in China, different levels of digital economy development, environmental regulation, and green development in various regions, the research results of the digital economy affecting green development at the national level may be quite different, so regional heterogeneity analysis was conducted. The 30 provinces (cities, districts) of the research object were divided into the east, the middle, and the west, and the sample areas were shared as shown in Table 7.

#### 5.3.1. Test on the Influence of the Digital Economy on Green Development in East China

To explore the influence of digital economy on green development in eastern China, we carried out the causality test. The regression results are shown in Table 8. Column (1) demonstrates the direct effect of the digital economy on green development. The digital economy has promoted green development, but the result is not significant. The possible reason is that the economic development quality in the eastern region is high, green production and development have been realized, and the influence of the digital economy on green development has begun to enter the stage of diminishing marginal benefits. Column (2) describes the influence of environmental regulation on green development. Environmental regulation has a restraining effect on green development, that is to say, with resource consumption and environmental pollution becoming more and more serious, the ability of green development of eastern of China region needs to be further improved. Column (3) illustrates the inverted U-shaped influence trend of the digital economy on green development, and it is significant at the level of 1%. Column (4) shows the regulating role of environmental regulation in the process of the digital economy improving green development ability. There is a substitution effect between environmental regulation and the digital economy. With the aggravation of pollution, the ability of the digital economy to promote green development is insufficient.

#### 5.3.2. Test on the Influence of the Digital Economy on Green Development in Central China

Table 9 reports the impact of the digital economy on green development in central China. Column (1) shows that the digital economy has a promoting impact on green development, but the result is not significant. In column (2), environmental regulation has an inhibitory effect on green development. Column (3) shows the inverted U-shaped influence trend of the digital economy on green development. In column (4), environmental regulation plays a regulatory role in the process of improving the green development capability of the digital economy. The reason may be that the development of the digital economy in the central region of China is in the rising period of fierce competition, and environmental regulation can promote green development. There is a synergistic effect between the digital economy and environmental regulation. With the enhancement of government environmental regulation means, the ability of the digital economy to promote green development will gradually increase.

#### 5.3.3. Test on the Influence of the Digital Economy on Green Development in Western China

Table 10 reports the impact of the digital economy on green development in western China. The results in column (1) show that the digital economy has a direct contributing effect on the level of green development. In the second column, environmental regulation has an inhibitory effect on green development, especially at the level of 1%. This is because the western region is mostly in a resource consumption-oriented economic development model, and high-intensity pollution of resources and the environment is not conducive to the enhancement of green development. In column (3), the digital economy has an inverted U-shaped influence trend on green development, and the regression coefficient has passed the significance level test of 1%. In column (4), environmental regulation plays a synergistic regulatory role in the process of promoting the green development of the digital economy. The possible reason may be that the economic level of the western region is slower than that of the eastern and central regions. The positive effects of the digital economy, such as infrastructure construction and human capital introduction, are conducive to technological innovation and knowledge dissemination in the western resource-based regions, while government environmental regulations can effectively control enterprises’ pollution emissions and innovative technology adoption. Under the regulation of environmental regulation, the combined effect of them is more significant in enhancing green development.

### 5.4. Robustness Test

#### 5.4.1. Changing the Calculation Method of the Green Development Index

To test the stability of the results and the reliability of the indicators, this paper changes the calculation method of the green development index in place of the green development level mentioned above. The regression results are shown in columns (1), (2), (3), and (4) in Table 11. After replacing the explained variables, the main variables in the regression results of the model are all significant, which is consistent with the previous article. The conclusions of the study are strongly robust.

#### 5.4.2. Changing the Research Sample

The research object includes four municipalities directly under the central government. Different levels of government environmental regulations may have great differences, resulting in great deviations in the role of the digital economy on green development. Therefore, the samples from Beijing, Tianjin, Shanghai, and Chongqing were discarded, while the samples from the remaining 26 provinces were maintained for statistical analysis. The results are shown in Table 12. The digital economy plays a significant role in promoting green growth. The results and conclusions are the same as above.

#### 5.4.3. Robustness Test of Instrumental Variable Method

Considering the endogenous problem, the lag phase of the digital economy is selected as the instrumental variable, and the two-stage least square method is adopted for regression. The regression results of the second stage are shown in column (5) of Table 12. It can be considered that the positive effect of the digital economy on green development still exists.

### 5.5. The Test of the Intermediary Effect of Technological Innovation on the Promotion of Green Development of the Digital Economy

We used a stepwise mediating effects model to investigate the role of technological innovation in the transmission mechanism of the digital economy’s impact path on green development, and the findings are shown in Table 13. Column (1) is the step-by-step mediation model test result, from which it can be seen the regression coefficient is at the level of 1% with the result of 0.1825, indicating that the digital economy can significantly improve the level of technological innovation. Column (2) is a benchmark regression for comparison. Column (3) is the regression result with intermediate variables. Although the coefficient of 8.1351 is lower than 11.2659 in column (2), which is still significantly positive, it shows that technological innovation plays a partial intermediary role in the digital economy, promoting green development; assuming H4 is tested.

The transmission path of the digital economy influencing green development lies in the following: firstly, digital technology usually has technical attributes, which can trigger and accelerate the technological innovation and diffusion of enterprise innovation subjects among departments and links such as production, sales, circulation, etc., and empower enterprise digital transformation and industrial structure adjustment and optimization. On the other hand, the collaborative interaction of innovation subjects can also promote knowledge spillovers and technical exchanges, strengthen regional cooperation and trade, and drive the technological innovation level in the entire region. Technological innovation, as the main measure of enterprise energy saving and emission reduction, can improve the utilization efficiency of labor, capital, and energy, reduce energy consumption and pollution emissions, and then improve the development level of the regional green economy.

## 6. Conclusions

This paper has examined the impact of the digital economy on green development and its mechanism based on measuring the development level of the digital economy and greening index on the basis of China’s provincial panel data from 2011 to 2019. According to the report, the greening degree of economic growth, resource and environmental carrying capacity, and government policy support are the three dimensions of the digital economy that directly contribute to green development. The regression coefficient of the digital economy on green development is 16.3204, indicating that for every 1% change in the digital economy index, the level of green development increases by 16.3204%. The digital economy influences the green development in an inverted U-shaped. The regression coefficient of the digital economy on green development is 73.7503, and the regression coefficient of the quadratic term of the digital economy on green development is −6.4 × 10^3^ ***, indicating that as the development capacity of the digital economy increases, the level of green development first rises and then gradually decreases. Environmental regulation is an effective regulatory variable, and environmental regulation has a substitution effect in the digital economy, affecting green development. The regression coefficient of the digital economy on green development is 18.7761, indicating that for every 1% increase in the digital economy, the level of green development increases by 18.7761%. The coefficient of the interaction term between the digital economy and environmental regulation is −0.8661, indicating that pollution emissions inhibit the contribution of the digital economy to green development. The digital economy drives green development with obvious regional heterogeneity. The regression coefficients of the regional digital economy influencing green development in the east, central and west are 9.8508, 11.2863 and 16.5660 respectively; the digital economy in the central and west regions has a stronger effect on enhancing green development. The analysis of the mechanism of action shows that the coefficient of the digital economy on green development is 8.1351, indicating that technological innovation plays a partial intermediary role in the promotion of green development by the digital economy. The robustness test has been conducted by changing the calculation method of the green development index, changing the research sample, and the instrumental variable method, and the conclusions are still valid.

Based on theoretical mechanisms and empirical test results, to strengthen the important role of the digital economy in driving green development, this paper proposes the following suggestions.

First, the layout and construction of digital infrastructure as well as the development and application of digital technology according to local conditions will improve the development level of the regional digital economy. China’s eastern, central and western regions have great differences in the economic foundation and digital development degree, and the quality level of economic development is also unbalanced and inadequate. According to the resource endowment and development requirements, it is necessary to appropriately layout new infrastructures such as 5G communication technology and data centers, enhance the level of regional digital construction, accelerate the implementation of the strategic layout of digital economy development, and highlight the leading role and continuous benign influence of digital economy on the green development of the regional economy.

Second, the government should take appropriate environmental regulation policies to intervene in the market, and urge enterprises to green production and upgrade industrial structure. In China’s long-term economic development, environmental protection policies mostly emphasize investment in environmental-related projects, thus promoting economic development. Nowadays, under the new development pattern, it is required to attach importance to environmental protection and ecological restoration, which requires the government to strengthen publicity and correct guidance, constantly improve the awareness of the public and enterprises on environmental protection and sustainable development, make work arrangements in the circular economy, low-carbon economy, energy conservation, and emission reduction, and carry out effective control. On the other hand, we should pay attention to and coordinate the regulatory effect of environmental regulation on the digital economy, and speed up the improvement of the government’s governance system for green development.

Third, attach importance to and give full play to the role of technological innovation, and essentially seek the sustainable driving force of the digital economy to promote the green development of the economy through technological innovation. Through the development and application of digital technology, with the help of digital transformation, the configuration of hardware facilities and the supporting environment for development will be changed. Relying on digital technology will promote rapid, low-cost, real-time information iteration, technology diffusion of relevant knowledge, and regional technological progress as well technological innovation. On the other hand, enterprises should increase the investment of internal R and D funds and scientific and technological human capital, strengthen the integration and application ability of various innovative resources, and realize green production and circulation. By formulating feasible schemes for digital economy, driving green development that fits the practical needs of regional development, the important role of digital economy for sustainable development will be fully utilized.

The following innovations and contributions are made by this article: Firstly, it puts out the theoretical hypothesis that the digital economy has an impact on how the economy develops sustainably, based on an existing literature study and economic theory. According to the findings of previous studies, this paper uses the method of index systems to measure and quantify the growth of the digital economy and environmental development in China’s provinces. It also expounds on and tests the role of direction and influence degree of the digital economy composite index on economic greening and its sub-dimensions. Secondly, the role of government oversight on the impact of the digital economy on green development is considered, and the moderating role of environmental regulation is examined. Thirdly, an intermediary effect model is proposed for empirical analysis in order to analyze the course of the digital economy’s influence on economic greening.

The article may have the following research deficiencies: Firstly, this study uses provincial panel data, and the urban panel data can be used to enrich the sample size in subsequent studies. Secondly, the topic of this thesis is to study the impact of the digital economy on regional green development from a macroeconomic perspective. Subsequent research can be carried out from the perspective of meso-industrial and micro-enterprise.

## Figures and Tables

**Table 1:** Evaluation index system of comprehensive development level of the digital economy.

Secondary Index	Index Measure	Weight
Internet penetration	Internet users/resident population at the end of the year	0.2108
employees in the information service industry	information, software, and information technology service industry employed persons in urban units/employed persons in urban units	0.1928
Internet-related output	total telecom business per capita (10,000 yuan)	0.1973
number of mobile internet users	number of mobile phones per 100 people (units)	0.2109
digital inclusive finance development	digital inclusive finance index	0.1882

**Table 2:** Descriptive statistics of variables.

Variable Type	Variable Name	Symbol	Mean	Sd	Min	Max
Explained variable	green development	GREEN	0.3805	0.0699	0.2810	0.6380
	greening degree of economic growth	ECO	0.1070	0.0453	0.0460	0.2560
	environmental carrying potential	ENV	0.0971	0.0304	0.0420	0.1790
	government policy support	GOV	0.1765	0.0395	0.0980	0.2940
explanatory variable	digital economy	DIG	0.0051	0.0018	0.0014	0.0095
regulated variable	environmental regulation	REG	0.5190	0.5273	0.0000	2.1487
mediator variable	technical innovation	INN	0.0109	0.0060	0.0020	0.0303
control variable	rationalize the structure of production	INR	0.5135	0.2836	−0.0580	1.1930
	marketization level	MAR	6.7243	1.9716	2.5500	10.9200
	urbanization level	URB	0.5783	0.1222	0.3680	0.8930
	government scale	SCAL	0.2487	0.1021	0.1189	0.6121
	openness to the outside world	OPEN	0.0202	0.0150	0.0004	0.0737
	human capital level	HUM	9.1673	0.8906	7.6094	12.6651

**Table 3:** Test of the direct effect of the digital economy on green development.

	(1)	(2)	(3)	(4)
	GREEN	ECO	ENV	GOV
DIG	16.3204 ***	5.4400 *	−3.8639 *	15.8743 ***
	(3.1050)	(1.8914)	(−1.8167)	(4.1039)
INR	−0.0431	−0.0427	0.0224	−0.0257
	(−0.8123)	(−1.4708)	(1.0444)	(−0.6581)
MAR	−0.0041	−0.0038	0.0033	−0.0047
	(−0.5609)	(−0.9502)	(1.1119)	(−0.8805)
URB	−0.8381 ***	−0.3273 ***	0.2021 **	−0.7625 ***
	(−4.3110)	(−3.0768)	(2.5690)	(−5.3291)
SCAL	0.1878	−0.0732	0.2129 ***	0.0701
	(1.3916)	(−0.9905)	(3.8973)	(0.7060)
OPEN	0.9872 ***	0.4665 **	−0.2742 *	0.8287 ***
	(2.7530)	(2.3771)	(−1.8898)	(3.1403)
HUM	−0.0446 ***	−0.0136	0.0075	−0.0362 ***
	(−2.6850)	(−1.4926)	(1.1079)	(−2.9563)
_CONS	1.1691 ***	0.4475 ***	−0.1490 *	0.8754 ***
	(5.7191)	(4.0004)	(−1.8015)	(5.8188)
N	270	270	270	270
R^2^	0.1311	0.1004	0.1511	0.1685

Note: Z values are in brackets. “*”, “**” and “***” are significant at the statistical level of 10%, 5% and 1% respectively.

**Table 4:** The direct impact of environmental regulation on green development.

	(1)	(2)	(3)	(4)
	GREEN	ECO	ENV	GOV
REG	−0.0465 ***	−0.0288 ***	0.0111	−0.0286 **
	(−2.7754)	(−3.1431)	(1.6204)	(−2.2565)
INR	−0.0808	−0.0555 *	0.0309	−0.0616
	(−1.5476)	(−1.9480)	(1.4457)	(−1.5619)
MAR	0.0039	−0.0026	0.0021	0.0037
	(0.6283)	(−0.7490)	(0.8359)	(0.7739)
URB	−0.3692 **	−0.1362 *	0.0719	−0.3195 ***
	(−2.4640)	(−1.6649)	(1.1697)	(−2.8215)
SCAL	0.3089 **	−0.0272	0.1781 ***	0.1913 *
	(2.3861)	(−0.3842)	(3.3556)	(1.9555)
OPEN	0.4497	0.2093	−0.1051	0.3344
	(1.3079)	(1.1149)	(−0.7458)	(1.2870)
HUM	−0.0328 **	−0.0133	0.0084	−0.0263 **
	(−2.1535)	(−1.5977)	(1.3472)	(−2.2865)
_CONS	0.8484 ***	0.3712 ***	−0.1000	0.5704 ***
	(5.2400)	(4.1998)	(−1.5064)	(4.6618)
N	270	270	270	270
R^2^	0.1231	0.1215	0.1459	0.1255

Note: Z values are in brackets. “*”, “**” and “***” are significant at the statistical level of 10%, 5% and 1% respectively.

**Table 5:** Nonlinear Effect Test of Digital Economy on Green Development.

	(1)	(2)	(3)	(4)
	GREEN	ECO	ENV	GOV
DIG	73.7503 ***	36.6389 ***	−28.5217 ***	65.2929 ***
	(10.4298)	(9.4709)	(−10.1007)	(13.6449)
DIG2	−6.4 × 10^3^ ***	−3.5 × 10^3^ ***	2.8 × 10^3^ ***	−5.5 × 10^3^ ***
	(−9.9417)	(−9.8468)	(10.8611)	(−12.7518)
REG	−0.0241 *	−0.0152 **	−0.0004	−0.0091
	(−1.7172)	(−1.9841)	(−0.0692)	(−0.9578)
INR	−0.0190	−0.0300	0.0100	−0.0027
	(−0.4336)	(−1.2542)	(0.5735)	(−0.0913)
MAR	0.0033	0.0001	−0.0003	0.0021
	(0.5432)	(0.0399)	(−0.1431)	(0.5175)
URB	−0.7676 ***	−0.2895 ***	0.1695 ***	−0.6993 ***
	(−4.8068)	(−3.3135)	(2.6584)	(−6.4708)
SCAL	0.1735	−0.0831	0.2085 ***	0.0692
	(1.5543)	(−1.3607)	(4.6786)	(0.9163)
OPEN	0.5120 *	0.2046	−0.0887	0.4399 **
	(1.7196)	(1.2560)	(−0.7461)	(2.1830)
HUM	−0.0359 ***	−0.0091	0.0025	−0.0274 ***
	(−2.6070)	(−1.2051)	(0.4598)	(−2.9367)
_CONS	0.9058 ***	0.3091 ***	−0.0123	0.6231 ***
	(5.2071)	(3.2484)	(−0.1771)	(5.2935)
N	267	267	267	267
R^2^	0.4204	0.4002	0.4485	0.5310

Note: Z values are in brackets. “*”, “**” and “***” are significant at the statistical level of 10%, 5% and 1% respectively.

**Table 6:** Regulation effect of the digital economy and environmental regulation on green development.

	(1)	(2)	(3)	(4)
	GREEN	ECO	ENV	GOV
DIG	18.7761 ***	8.4662 ***	−4.2682 *	15.8988 ***
	(3.4059)	(2.8369)	(−1.8841)	(3.9045)
REG	−0.0479 *	−0.0122	0.0127	−0.0464 **
	(−1.9533)	(−0.9150)	(1.2581)	(−2.5587)
DIG*REG	−0.8661	−3.1531 *	−0.0707	2.0322
	(−0.2831)	(−1.9042)	(−0.0562)	(0.8995)
INR	−0.0496	−0.0384	0.0248	−0.0378
	(−0.9358)	(−1.3382)	(1.1365)	(−0.9659)
MAR	−0.0064	−0.0062	0.0037	−0.0052
	(−0.8789)	(−1.5761)	(1.2367)	(−0.9592)
URB	−0.8364 ***	−0.2938 ***	0.2050 **	−0.7932 ***
	(−4.2981)	(−2.7890)	(2.5640)	(−5.5183)
SCAL	0.1378	−0.0966	0.2250 ***	0.0323
	(1.0311)	(−1.3348)	(4.0962)	(0.3269)
OPEN	0.8772 **	0.3903 **	−0.2500 *	0.7694 ***
	(2.4783)	(2.0373)	(−1.7191)	(2.9430)
HUM	−0.0509 ***	−0.0195 **	0.0087	−0.0379 ***
	(−3.0599)	(−2.1689)	(1.2673)	(−3.0892)
_CONS	1.2738 ***	0.5035 ***	−0.1737 **	0.9478 ***
	(6.2596)	(4.5707)	(−2.0774)	(6.3060)
N	267	267	267	267
R^2^	0.1695	0.1585	0.1632	0.1994

Note: Z values are in brackets. “*”, “**” and “***” are significant at the statistical level of 10%, 5% and 1% respectively.

**Table 7:** Division of sample areas.

Zone	Province
eastern	Beijing, Tianjin, Hebei, Liaoning, Shanghai, Jiangsu, Zhejiang, Fujian, Shandong, Guangdong, and Hainan
middle	Shanxi, Jilin, Heilongjiang, Anhui, Jiangxi, Henan, Hubei, and Hunan
west	Inner Mongolia, Guangxi, Chongqing, Sichuan, Guizhou, Yunnan, Shaanxi, Gansu, Qinghai, Ningxia, and Xinjiang

**Table 8:** Test results of the influence of the digital economy on green development in eastern China.

	(1)	(2)	(3)	(4)
	GREEN	GREEN	GREEN	GREEN
DIG	9.8508		112.1642 ***	12.2264
	(1.1762)		(8.1127)	(1.3315)
REG		−0.0226	−0.0124	−0.0321
		(−0.7856)	(−0.5640)	(−0.6231)
DIG2			−8.1 × 10^3^ ***	
			(−8.1561)	
DIG*REG				−0.1005
				(−0.0159)
INR	−0.0128	−0.0087	−0.0104	0.0316
	(−0.0660)	(−0.0445)	(−0.0712)	(0.1556)
MAR	−0.0144	−0.0056	−0.0096	−0.0141
	(−1.1130)	(−0.4874)	(−1.0018)	(−1.0848)
URB	−0.7136 *	−0.4147	−1.3421 ***	−0.6987
	(−1.9532)	(−1.3632)	(−4.7647)	(−1.4893)
SCAL	0.7078 **	0.7073 *	0.3525	0.6285 *
	(2.0048)	(1.9780)	(1.3108)	(1.7180)
OPEN	0.5255	0.3273	1.0638 ***	0.4886
	(1.1171)	(0.7117)	(2.9860)	(1.0248)
HUM	−0.0581 *	−0.0395	−0.0597 **	−0.0611 *
	(−1.7404)	(−1.3176)	(−2.4058)	(−1.7506)
_CONS	1.4072 ***	1.0261 **	1.5793 ***	1.4391 **
	(2.6964)	(2.3641)	(4.0761)	(2.6394)
N	99	99	99	99
R^2^	0.1442	0.1362	0.5425	0.1572

Note: Z values are in brackets. “*”, “**” and “***” are significant at the statistical level of 10%, 5% and 1% respectively.

**Table 9:** Test results of the influence of the digital economy on green development in central China.

	(1)	(2)	(3)	(4)
	GREEN	GREEN	GREEN	GREEN
DIG	11.2863		63.1352 ***	3.2171
	(1.2811)		(5.3511)	(0.3523)
REG		−0.0312	−0.0091	−0.1046 **
		(−1.2871)	(−0.4601)	(−2.4909)
DIG2			−7.9 × 10^3^ ***	
			(−5.6068)	
DIG*REG				17.1216 **
				(2.1861)
INR	0.0101	0.0137	0.0449	0.0635
	(0.1208)	(0.1646)	(0.6681)	(0.7505)
MAR	−0.0010	0.0090	0.0035	0.0065
	(−0.0568)	(0.5703)	(0.2483)	(0.3823)
URB	−0.7510 *	−0.4679	0.2636	−0.9084 **
	(−1.9064)	(−1.4298)	(0.7276)	(−2.3303)
SCAL	−0.0146	0.0551	−0.0719	0.0175
	(−0.0702)	(0.2791)	(−0.4313)	(0.0871)
OPEN	5.4529 ***	5.7460 ***	2.5191 *	7.1004 ***
	(3.3927)	(3.6044)	(1.8052)	(4.1418)
HUM	0.0148	0.0229	0.0048	0.0123
	(0.4631)	(0.7544)	(0.1850)	(0.3966)
_CONS	0.4377	0.1901	−0.0252	0.4717
	(1.1748)	(0.6689)	(−0.0807)	(1.3082)
N	72	72	72	72
R^2^	0.2484	0.2486	0.5332	0.3251

Note: Z values are in brackets. “*”, “**” and “***” are significant at the statistical level of 10%, 5% and 1% respectively.

**Table 10:** Test results of the influence of the digital economy on green development in western China.

	(1)	(2)	(3)	(4)
	GREEN	GREEN	GREEN	GREEN
DIG	16.5660		58.4625 ***	5.0006
	(1.4724)		(3.8941)	(0.4207)
REG		−0.1259 ***	−0.0831 **	−0.2965 ***
		(−3.4446)	(−2.2900)	(−3.6317)
DIG2			−6.5 × 10^3^ ***	
			(−3.5176)	
DIG*REG				25.6300 **
				(2.2692)
INR	−0.1562	−0.2881 ***	−0.0707	−0.2621 ***
	(−1.5788)	(−3.4593)	(−0.7362)	(−2.7822)
MAR	0.0179	0.0218 *	0.0067	0.0221
	(1.2673)	(1.8110)	(0.5371)	(1.5983)
URB	−1.4098 ***	−1.0391 ***	−0.2950	−1.4020 ***
	(−3.5925)	(−3.3572)	(−0.6350)	(−3.9219)
SCAL	0.4132 **	0.5126 ***	0.3643 **	0.3749 **
	(2.1730)	(2.9897)	(2.1832)	(2.1523)
OPEN	−0.8637	−1.9355 *	−0.7627	−1.7136
	(−0.6757)	(−1.8094)	(−0.6818)	(−1.4136)
HUM	−0.0441 *	−0.0294	−0.0309	−0.0424 *
	(−1.7450)	(−1.2838)	(−1.3827)	(−1.8382)
_CONS	1.2644 ***	1.1266 ***	0.5919 *	1.4333 ***
	(4.4903)	(4.7464)	(1.8149)	(5.5019)
N	99	99	99	99
R^2^	0.2402	0.3195	0.4338	0.3852

Note: Z values are in brackets. “*”, “**” and “***” are significant at the statistical level of 10%, 5% and 1% respectively.

**Table 11:** Robustness test results of changing the calculation method of green development index.

	(1)	(2)	(3)	(4)
	GREEN2	GREEN2	GREEN2	GREEN2
DIG	3.5621 **		19.0968 ***	4.1258 ***
	(2.3713)		(8.8010)	(2.6163)
REG		−0.0127 **	−0.0074 *	−0.0134 *
		(−2.5818)	(−1.7049)	(−1.8328)
DIG2			−1.6 × 10^3^ ***	
			(−8.6473)	
DIG*REG				−0.1354
				(−0.1485)
INR	−0.0113	−0.0228	−0.0053	−0.0132
	(−0.7155)	(−1.4800)	(−0.3908)	(−0.8362)
MAR	−0.0013	0.0012	0.0005	−0.0019
	(−0.6175)	(0.6445)	(0.2438)	(−0.8851)
URB	−0.1953 ***	−0.1080 **	−0.2293 ***	−0.1945 ***
	(−3.5376)	(−2.4456)	(−4.8293)	(−3.4797)
SCAL	0.1035 ***	0.1205 ***	0.0812 **	0.0905 **
	(2.6143)	(3.1594)	(2.3749)	(2.2956)
OPEN	0.2263 **	0.1210	0.1614 *	0.1964 *
	(2.1566)	(1.1950)	(1.7842)	(1.8855)
HUM	−0.0123 **	−0.0085 *	−0.0094 **	−0.0138 ***
	(−2.5188)	(−1.8978)	(−2.2335)	(−2.8185)
_CONS	0.3156 ***	0.2425 ***	0.2719 ***	0.3425 ***
	(5.2615)	(5.0836)	(5.1572)	(5.7120)
N	270	270	270	270
R^2^	0.1160	0.1199	0.3557	0.1472

Note: Z values are in brackets. “*”, “**” and “***” are significant at the statistical level of 10%, 5% and 1% respectively.

**Table 12:** Delete the robustness test of municipalities directly under the Central Government.

	(1)	(2)	(3)	(4)	(5)
	GREEN	GREEN	GREEN	GREEN	GREEN
DIG	18.2825 ***		69.1434 ***	20.6133 ***	−15.1874 ***
	(3.0147)		(9.7218)	(3.2401)	(−4.7294)
L.DIG					0.8362 ***
					(74.3104)
REG		−0.0477 ***	−0.0212	−0.0475 **	
		(−2.9449)	(−1.5630)	(−1.9867)	
DIG2			−7.1 × 10^3^ ***		
			(−9.5832)		
DIG*REG				−0.8366	
				(−0.2732)	
INR	−0.0859	−0.1294 **	−0.0356	−0.0946 *	−0.0137
	(−1.5628)	(−2.3869)	(−0.7909)	(−1.7332)	(−0.5117)
MAR	−0.0013	0.0105	0.0108	−0.0026	0.0019
	(−0.1666)	(1.4736)	(1.6095)	(−0.3280)	(0.3450)
URB	−1.1558 ***	−0.6321 ***	−0.4463 **	−1.1648 ***	0.3621 ***
	(−4.7668)	(−3.5248)	(−2.1218)	(−4.8972)	(4.2723)
SCAL	0.2967 **	0.3464 **	0.2023 *	0.2346 *	0.0621
	(2.0992)	(2.4978)	(1.7510)	(1.6803)	(0.8681)
OPEN	0.9677 **	0.1336	−0.2842	0.7643 *	0.0669
	(2.0914)	(0.3148)	(−0.7221)	(1.6696)	(0.2204)
HUM	−0.0265	−0.0175	−0.0347 **	−0.0323 *	0.0105
	(−1.5365)	(−1.0425)	(−2.4692)	(−1.8874)	(0.9910)
_CONS	1.1083 ***	0.8135 ***	0.6917 ***	1.2159 ***	0.1374
	(5.4473)	(4.9026)	(3.9371)	(6.0260)	(1.5863)
N	234	234	234	234	240
R^2^	0.1485	0.1468	0.4475	0.1929	0.420

Note: Z values are in brackets. “*”, “**” and “***”are significant at the statistical level of 10%, 5% and 1% respectively.

**Table 13:** Step-by-step intermediary effect test.

	(1)	(2)	(3)
	INN	GREEN	GREEN
DIG	0.1825 ***	11.2659 **	8.1351 *
	(2.9019)	(2.3674)	(1.8235)
INN			12.4604 ***
			(7.0897)
INR	0.0055 ***	−0.0466	−0.1150 *
	(2.9750)	(−1.0660)	(−1.8268)
MAR	−0.0000	−0.0048	−0.0046
	(−0.0732)	(−0.7576)	(−0.5902)
URB	0.0255 ***	−0.6565 ***	−0.9745 ***
	(3.9892)	(−3.2458)	(−4.3587)
SCAL	−0.0176 ***	0.2923 *	0.5121 ***
	(−3.8206)	(1.8030)	(3.7514)
OPEN	0.0972 ***	0.7831	−0.4277
	(7.9389)	(1.6069)	(−0.9673)
HUM	−0.0012 **	−0.0432 **	−0.0279
	(−2.1643)	(−2.1114)	(−1.4460)
_CONS	0.0059	1.0671 ***	0.9937 ***
	(0.8426)	(4.3527)	(3.9755)
N	270	270	270
R^2^	0.3410	0.1115	0.2670

Note: Z values are in brackets. “*”, “**” and “***” are significant at the statistical level of 10%, 5% and 1% respectively.

## Data Availability

Not applicable.

## References

[1] Shao S., Zhang K., Dou J. (2019). Energy saving and emission reduction effect of economic agglomeration: Theory and Chinese experience. Manag. World.

[2] Jbaily A., Zhou X., Liu J., Lee T.H., Kamareddine L., Verguet S., Dominici F. (2022). Air pollution exposure disparities across US population and income groups. Nature.

[3] He W., Wen J., Zhang M. (2022). Research on the influence of digital economy development on China’s green ecological efficiency based on the two-way fixed effect model. Econ. Issues.

[4] Qu G., Yang L., Qu W., Li Q. (2021). Evolutionary game study considering the choice of government regulation and public supervision strategies under the third-party international environmental audit. China Manag. Sci..

[5] Qu G., Zhang Z., Li C., Qin K., Qu W., Xu Y., Zhou X. (2021). Triangular fuzzy game analysis considering public participation in third-party international environmental audit. Oper. Res. Manag..

[6] Qiu L., Zhou M., Wei X. (2018). Regulation, innovation, and firm selection: The porter hypothesis under monopolistic competition. J. Environ. Econ. Manag..

[7] Qu G., Zhang H., Liu Z., Xu L., Zhang Z., Zhang Q. (2015). Analysis based on environmental information and financial market asymmetric game model. China Manag. Sci..

[8] Jonathan C., Ian H., Jay S., John V. (2020). Disparities in PM2.5 air pollution in the United States. Science.

[9] Qu G., Liu X., Li Y., Qu W., Li S., Zhang Q. (2020). Fuzzy game analysis of government regulation and enterprises’ participation in third-party international environmental audit. China Manag. Sci..

[10] Harding T., Herzberg J., Kuralbayeva K. (2021). Commodity prices and robust environmental regulation: Evidence from deforestation in Brazil. J. Environ. Econ. Manag..

[11] Li X. (2019). New features of digital economy and formation mechanism of the new kinetic energy of digital economy. Reform.

[12] Chen H., Shi Y., Xu M., Xu Z., Zou W. (2022). China’s industrial green development and its influencing factors under the background of carbon neutrality. Environ. Sci. Pollut. Res..

[13] Li Z., Wang J. (2022). The Dynamic Impact of Digital Economy on Carbon Emission Reduction: Evidence City-level Empirical Data in China. J. Clean. Prod..

[14] Wang L., Chen L., Li Y. (2022). Digital economy and urban low-carbon sustainable development: The role of innovation factor mobility in China. Environ. Sci. Pollut. Res. Int..

[15] Yu H., Zhu Q. (2022). Impact and mechanism of digital economy on China’s carbon emissions: From the perspective of spatial heterogeneity. Environ. Sci. Pollut. Res. Int..

[16] Zhang H. (2022). Pathways to carbon neutrality in major exporting countries: The threshold effect of digital transition. Environ. Sci. Pollut. Res. Int..

[17] Jia L., Hu X., Zhao Z., He B., Liu W. (2022). How Environmental Regulation, Digital Development and Technological Innovation Affect China’s Green Economy Performance: Evidence from Dynamic Thresholds and System GMM Panel Data Approaches. Energies.

[18] He J. (2022). Economic and comprehensive energy performance evaluation and business model of power enterprises under the double carbon background. Electron. Commer. Res..

[19] Tapscott D. (1996). The Digital Economy: Promise and Peril in the Age of Networked Intelligence.

[20] Landefeld J.S., Fraumeni B.M. (2001). Measuring the New Economy. BEA Pap. 0011 Bur. Econ. Anal..

[21] Berisha-Shaqiri A., Berisha-Namani M. (2015). Information Technology, and the Digital Economy. J. Soc. Sci..

[22] Li G., Tao T. (2018). E-commerce platform ecology and platform governance policy. Manag. World.

[23] Bharadwaj P. (2013). Digital Business Strategy: Toward a Next Generation of Insights. MIS Q..

[24] Xu P., Xu X. (2020). Logic and Analysis Framework of Enterprise Management Change in the Age of Artificial Intelligence. Manag. World.

[25] Xu X., Zhang M. (2020). Research on the scale measurement of China’s digital economy based on the perspective of international comparison. China Ind. Econ..

[26] Xu Q., Shan Z., Ma C. (2018). Overview of Research on Digital Economy Measurement Index System at Home and Abroad. Res. World.

[27] Zhao T., Zhang Z., Liang S. (2020). Digital economy, entrepreneurial activity, and high-quality development-empirical evidence from Chinese cities. Manag. World.

[28] Liu J., Yang Y., Zhang S. (2020). Research on Measurement and Driving Factors of China’s Digital Economy. Shanghai Econ. Res..

[29] Zhou X., Liu Y., Peng L. (2021). Development of digital economy and improvement of green total factor productivity. Shanghai Econ. Res..

[30] Fan Y., Hao X. (2021). Can the development of China’s digital economy bring about economic greening?—Empirical evidence from China’s provincial panel data. Explor. Econ. Issues.

[31] Zhang J., Lyu Y., Li Y., Geng Y. (2022). Digital economy: An innovation driving factor for low-carbon development. Environ. Impact Assess. Rev..

[32] Pierce D., He X. (1996). Blueprint of Green Economy.

[33] Bartelmus P. (2013). The future we want: Green growth or sustainable development. Environ. Dev..

[34] Janicke M. (2012). “Green growth”, From a growing eco-industry to economic sustainability. Energy Policy.

[35] Hou W. (2004). Research on China’s Green Development in the 21st Century. Nandu Acad..

[36] Wang L., Zhang Y. (2012). A probe into the connotation of “green development”. Social. Stud..

[37] Hu A., Zhou S. (2014). Green Development: Function Definition Mechanism Analysis and Development Strategy. China Popul. Resour. Environ..

[38] Li X., Pan J. (2011). Compilation of China’s Green Development Index—A Brief Introduction of 2010 Annual Report of China’s Green Development Index—Inter-provincial Comparison. Econ. Res. Ref..

[39] Li Z., Ouyang Q. (2017). Current situation and countermeasures of greening development of urban agglomerations in the middle reach of the Yangtze River. J. Nantong Univ. (Soc. Sci. Ed.).

[40] Ramanathan R. (2006). A multi-factor efficiency perspective to the relationships among world GDP, energy consumption and carbon dioxide emissions. Technol. Forecast. Soc. Change.

[41] Wang X., Xu X. (2020). The temporal and spatial evolution of high-quality economic development in the Yangtze River Economic Belt and the regional gap. Econ. Geogr..

[42] Khan A., Wu X. (2022). Digital economy and environmental sustainability: Do Information Communication and Technology (ICT) and economic complexity matter?. Int. J. Environ. Res. Public Health.

[43] Cheng W., Qian X. (2021). Digital economy and China’s industrial green total factor productivity growth. Econ. Issues Explor..

[44] Hosan S., Karmaker S., Rahman M., Chapman A.J., Saha B.B. (2022). Dynamic links among the demographic dividend, digitalization, energy intensity, and sustainable economic growth: Empirical evidence from emerging economies. J. Clean. Prod..

[45] Shen Z., Wang S., Boussemart J., Hao Y. (2022). Digital transition and green growth in Chinese agriculture. Technol. Forecast. Soc. Change.

[46] Cao S., Nie L., Sun H., Sun W., Taghizadeh-Hesary F. (2021). Digital finance, green technological innovation and energy-environmental performance: Evidence from China’s regional economies. J. Clean. Prod..

[47] Zhang X., Wan G., Zhang J., He Z. (2019). Digital economy, inclusive finance, and inclusive growth. Econ. Res..

[48] Yuan Y., Xie R. (2015). FDI, environmental regulation, and China’s industrial green total factor productivity growth-an empirical study based on the Luenberger index. Int. Trade Issues.

[49] Muhammad S., Wang J., Dong K., Zhao J. (2022). The impact of the digital economy on energy transition across the globe: The mediating role of government governance. Renew. Sustain. Energy Rev..

[50] Miao Z. (2021). Digital economy value chain: Concept, model structure, and mechanism. Appl. Econ..

[51] Lange S., Santarius T., Pohl J. (2020). Digitalization and Energy Consumption. Does ICT Reduce Energy Demand?. Ecol. Econ..

[52] Criado C.O., Valente S., Stengos T. (2011). Growth and pollution convergence: Theory and evidence. J. Environ. Econ. Manag..

[53] Tang L.Y., Lu B.K., Tian T.H. (2021). Spatial correlation network and regional differences for the development of digital economy in China. Entropy.

[54] Antonio B., Matthew F., Corey L. (2015). Who benefits from environmental regulation? Evidence from the Clean Air Act Amendments. Rev. Econ. Stat..

[55] Liu M., Tan R., Zhang B. (2021). The costs of “blue sky”: Environmental regulation, technology upgrading, and labor demand in China. J. Dev. Econ..

[56] Fu S., Viard V.B., Zhang P. (2021). Air pollution and manufacturing firm productivity: Nationwide estimates for China. Econ. J..

[57] Sareen S., Haarstad H. (2021). Digitalization as a driver of transformative environmental innovation-ScienceDirect. Environ. Innov. Soc. Transit..

[58] Maiti M., Kayal P. (2017). Digitization: Its impact on economic development & trade. Asian Econ. Financ. Rev..

[59] Fanea-Ivanovici M., Muetescu R.C., Pan M.C. (2019). Fighting corruption and enhancing tax compliance through digital public services: A way to increase sustainable development in Romania. Sustainability.

[60] Joseph S.S., Reed W. (2018). Why is pollution from US manufacturing declining? The roles of environmental regulation, productivity, and trade. Am. Econ. Rev..

[61] Alwakid W., Aparicio S., Urbano D. (2021). The influence of green entrepreneurship on sustainable development in Saudi Arabia: The role of formal institutions. Int. J. Environ. Res. Public Health.

[62] Deng R., Zhang A. (2022). Research on the influence and mechanism of digital economy development on environmental pollution in Chinese cities. South. Econ..

[63] Zhang D., Vigne S. (2021). How does innovation efficiency contribute to green productivity? A financial constraint perspective. J. Clean. Prod..

[64] Zhang X. (2019). Research on the Evolution of Innovation Mode under the Digital Economy. Economist.

[65] Xu X., Ren X., Chang Z. (2019). Big Data and Green Development. China Ind. Econ..

[66] Feng Z., Cheng S., Jian S. (2021). Research on the Impact of China’s Industrial Restructuring on High Quality Economic Development. J. Nanjing Univ. Financ. Econ..

[67] Wen Z., Ye B. (2014). Analyses of Mediating Effects: The Development of Methods and Models. Prog. Psychol. Sci..

[68] Huang Q., Yu Y., Zhang S. (2019). Internet development and manufacturing productivity improvement: Internal mechanism and Chinese experience. China Ind. Econ..

[69] Guo F., Wang J., Wang F., Kong T., Zhang X., Cheng Z. (2020). Measuring the development of digital inclusive finance in China: Index compilation and spatial characteristics. Economics.

[70] Yang L., Sun Z. (2015). Evaluation of the development level of new urbanization in western China based on the entropy method. Econ. Issues.

[71] Ye Q., Zeng G., Dai S., Wang F. (2018). Effects of different environmental regulatory tools on technological innovation of energy conservation and emission reduction in China—Based on panel data of 285 prefecture-level cities. China Popul. Resour. Environ..

[72] Liu X., Kong F. (2021). Research on the influence of digital economy development in the Yangtze River Economic Belt on urban green transformation based on the perspective of “Sansheng” space. Contemp. Econ. Manag..

[73] Gan C., Zheng R., Yu D. (2011). The Impact of China’s Industrial Structure Change on Economic Growth and Fluctuation. Econ. Res..

[74] Wang X., Fan G., Hu L. (2020). Report on China’s Provincial Marketization Index (2019).

[75] Ding Y., Qin Z. (2021). The impact of information and communication technology on the efficiency of a green economy-an empirical study based on the panel Tobit model. Learn. Pract..

[76] Zhang C., Zhong C. (2021). Financial innovation, industrial structure change, and high-quality economic development. Jianghan Forum.

[77] Ji j., Xu Y., Zhang Y. (2021). Study on relationship between environmental regulation and green total factor productivity from the perspective of private investment. China Manag. Sci..

[78] Ye J. (2021). Test of a nonlinear relationship between environmental regulation and high-quality development of China’s economy. Econ. Empir. Res..

